# Supplementary material for: Selective Recognition of Gallic Acid Using Hollow Magnetic Molecularly Imprinted Polymers with Double Imprinting Surfaces
Source: Polymers (Basel). 2022 Jan 2;14(1):175. doi: 10.3390/polym14010175 (PMC8747617; doi:10.3390/polym14010175)
Supplement: Supplementary file 1 [file polymers-14-00175-s001.zip › polymers-1524796-supplementary.pdf]

Supplementary Material

Selective recognition of gallic acid using hollow magnetic molecularly imprinted polymers with double imprinting surfaces

Jiawei Li[a], Xinji Zhou[a], Yu Yan[a], Dianling Shen[a], Danqing Lu[a], Yaping Guo[a], Lianwu Xie[a, *], Bin Deng[b, *]

[a] *College of Sciences, Central South University of Forestry and Technology, Changsha 410004, P. R. China;*

[b] *College of Chemistry Biology and Environmental Engineering, Xiangnan University, Chenzhou 423043, P. R. China*

* Correspondence: xielianwu@csuft.edu.cn (L.W. Xie); ddhbyjs@126.com (B. Deng).

Legend of supplementary Tables and Figures:

Figure S1. MIP synthesis flowchart

Figure S2. Application of magnetic molecularly imprinted polymers.

Figure S3. Gallic acid and its structural analogues.

Figure S4 Measuring width of hollow in HMMIP.

Figure S5 The BET specific surface area measurements for HMMIP (A), MMIP (B), and MNIP (C).

Figure S6 The shrinkage of imprinted polymer (yellow part represents the surface of imprinted layer, white part represents mesopore channels, solid arrow represents the direction of wrinkle, and dotted arrow points to the end of wrinkling).

Figure S7. Vibrating sample magnetometer curves of Fe_3O_4, Fe_3O_4@$mSiO_2$, MMIP and HMMIP samples and the magnetic separation of HMMIP under external magnet.

Figure S8. TGA curves of MMIP and HMMIP.

Figure S9. Adsorption kinetics curves of HMMIP, MMIP and MNIP at 318 K (a); quasi-first-order kinetic equation fitting of HMMIP (b), MMIP (d), and MNIP (f); quasi-second-order kinetic equation fitting of HMMIP (c), MMIP (e), and MNIP (g).

Figure S10 Comparison of GA adsorption on HMMIP and MMIP from green tea solution by HPLC

Table S1. The ratio of mobile phase and detection wavelength for HPLC.

Table S2. Data of pore size and specific surface area.

Table S3. Simulation parameters of quasi-first-order and quasi-second-order dynamics equations for three kinds of microspheres at 318 K.

Table S4. Simulation parameters of Langmuir equation and Freundlich equation for adsorption thermodynamics of HMMIP, MMIP, and MNIP

Table S5. Isolate factors β of HMMIP and MMIP

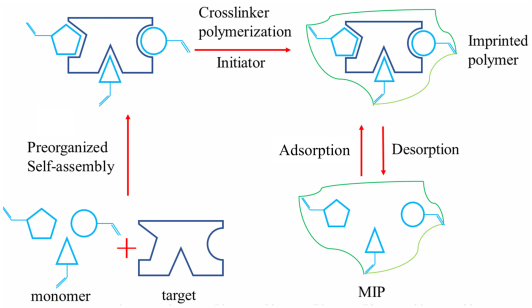

Figure S1. MIP synthesis flowchart.

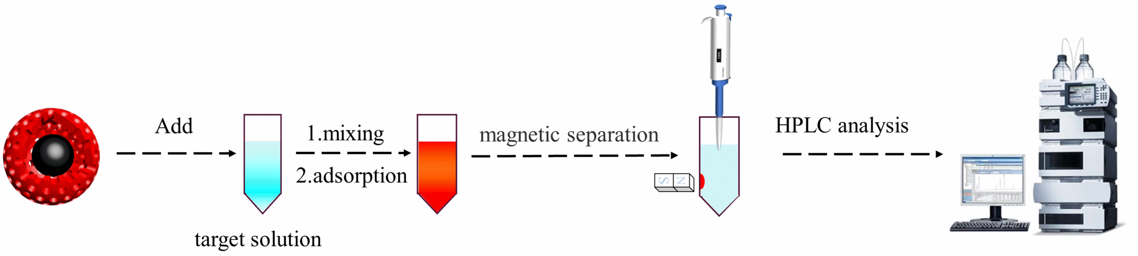

Figure S2. Application of magnetic molecularly imprinted polymers.

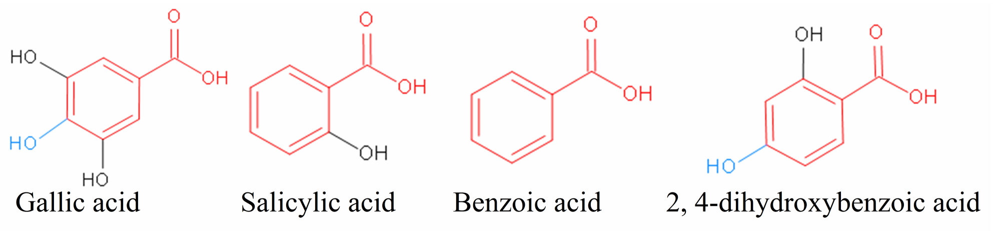

Figure S3. Gallic acid and its structural analogues.

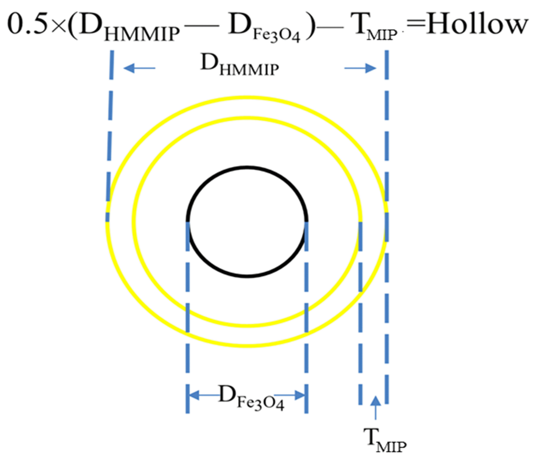

Figure S4. Measuring width of hollow in HMMIP .

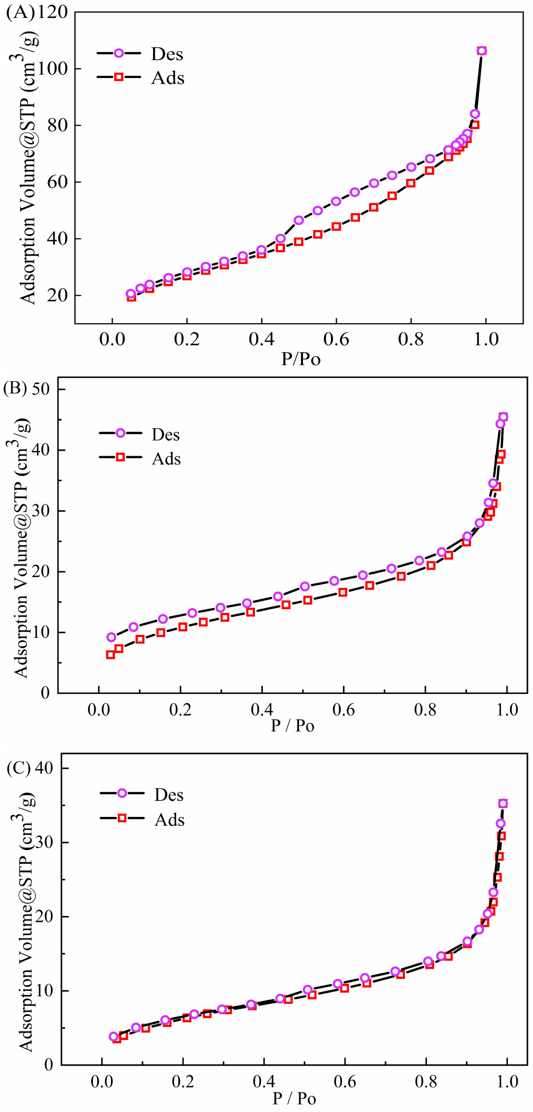

Figure S5. BET specific surface area measurements for HMMIP (A), MMIP (B), and MNIP (C).

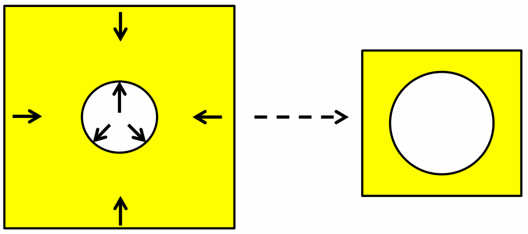

Figure S6. The shrinkage of imprinted polymer (yellow part represents the surface of imprinted layer, white part represents mesopore channels, solid arrow represents the direction of wrinkle, and dotted arrow points to the end of wrinkling).

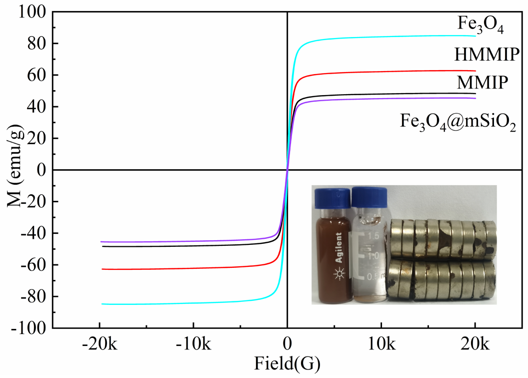

Figure S7. Vibrating sample magnetometer curves of Fe_3O_4, Fe_3O_4@mSiO_2, MMIP and HMMIP samples and the magnetic separation of HMMIP under an external magnet.

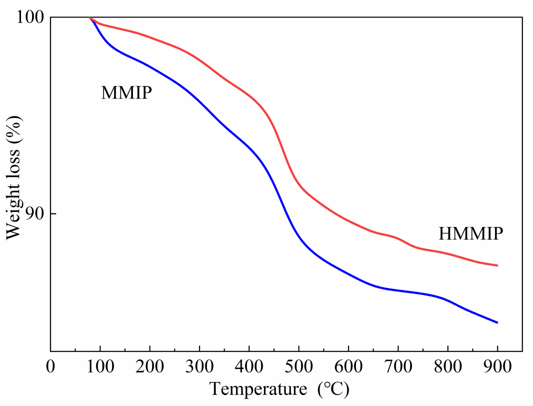

Figure S8. TGA curves of MMIP and HMMIP.

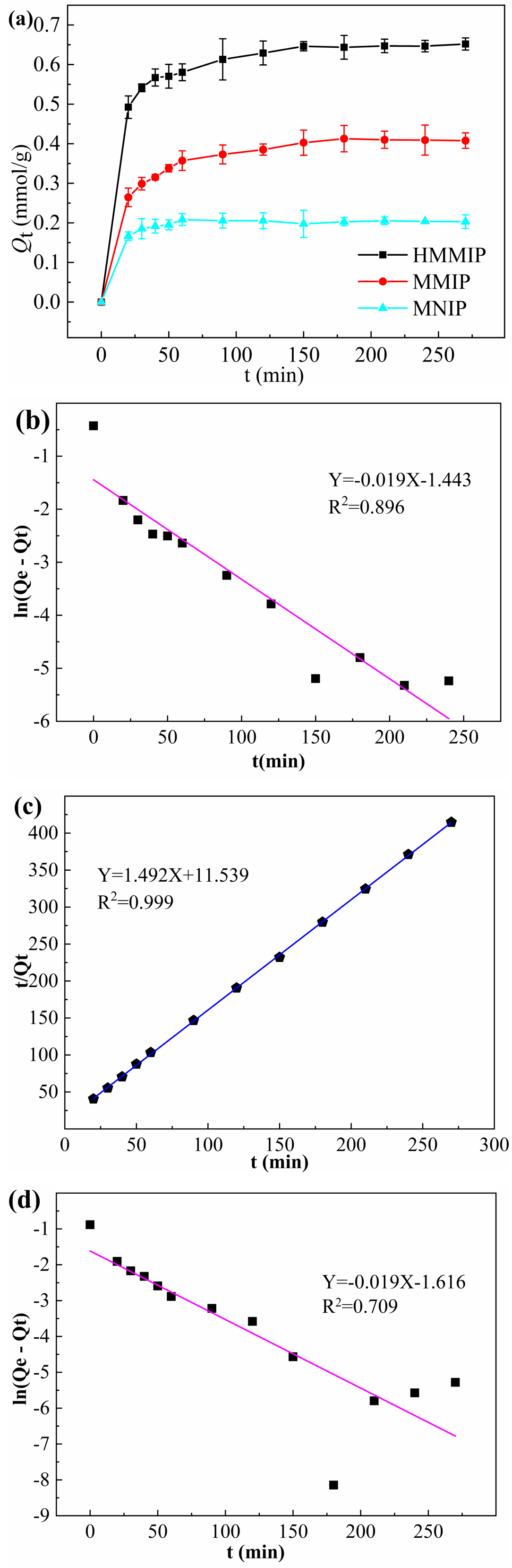

(a)
Q_t (mmol/g)
t (min)
HMMIP
MMIP
MNIP
(b)
ln(Qe - Qt)
t(min)
Y=-0.019X-1.443
R^2=0.896
(c)
t/Qt
t (min)
Y=1.492X+11.539
R^2=0.999
(d)
ln(Qe - Qt)
t (min)
Y=-0.019X-1.616
R^2=0.709

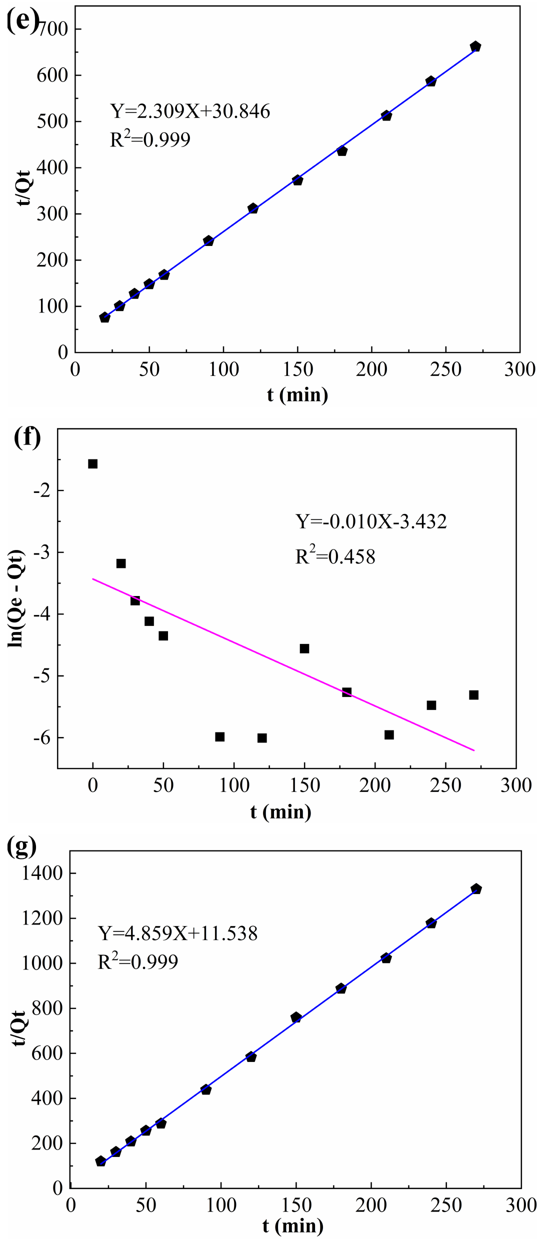

Figure S9. Adsorption kinetics curves of HMMIP, MMIP and MNIP at 318 K (a); quasi-first-order kinetic equation fitting of HMMIP (b), MMIP (d), and MNIP (f); quasi-second-order kinetic equation fitting of HMMIP (c), MMIP (e), and MNIP (g).

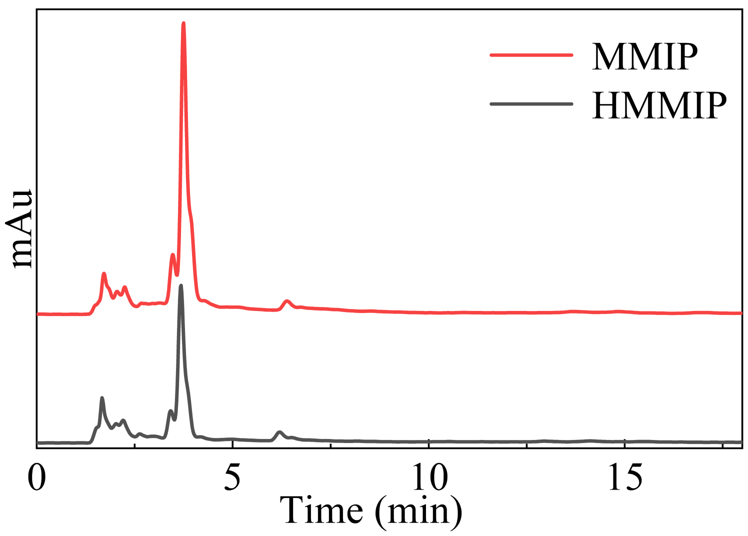

Figure S10. Comparison of gallic acid adsorption on HMMIP and MMIP from green tea solution by HPLC.

Table S1. The ratio of mobile phase and detection wavelength for HPLC.

Analytes	Wavelength (nm)	Mobile phase A	Mobile phase B	A:B (V/V)
Gallic acid	270	0.1% phosphoric acid in H_2O	methanol	85:15
Salicylic acid	230	0.1% phosphoric acid in H_2O	methanol	55:45
Benzoic acid	230	0.1% phosphoric acid in H_2O	methanol	55:45
2.4-dihydroxybenzoic acid	280	3% glacial acetic acid in H_2O	methanol	75:25

Table S2. Data of pore size and specific surface area.

	Average pore Diameter (nm)	Total pore volume (cm^3/g)	Surface Area (m^2/g)
MNIP	3.80	0.048	23.79
MMIP	3.82	0.057	39.06
HMMIP	6.74	0.165	97.59

Table S3. Simulation parameters of quasi-first-order and quasi-second-order dynamics equations for three kinds of microspheres at 318 K.

**Adsorbents**	$\boldsymbol{Q_e}$**(exp)** (mmol/g)	**Pseudo-first-order**			**Pseudo-second-order**		
		$\boldsymbol{Q_e}$ (mmol/g)	$\mathbf{k_1}$ (min^{-1})	$\mathbf{R^2}$	$\boldsymbol{Q_e}$ (mmol/g)	$\mathbf{k_2}$ (g mg^{-1} min^{-1})	$\mathbf{R^2}$
HMMIP	0.652	0.236	-0.019	0.896	0.670	0.129	0.999
MMIP	0.413	0.198	-0.019	0.709	0.433	0.075	0.999
MNIP	0.208	0.032	-0.010	0.458	0.206	0.421	0.999

Table S4. Simulation parameters of Langmuir equation and Freundlich equation for adsorption thermodynamics of HMMIP, MMIP, and MNIP

MIP	T (K)	Q_e(exp) (mmol /g)	Langmuir			Freundlich		
			Q_m(cal) (mmol /g)	K_L	R_1^2	K_F (mmol /g)	m	R_2^2
	318	2.815±0.054	2.593	0.387	0.9991	0.510	0.834	0.9553
HMMIP	308	1.384±0.022	0.778	1.302	0.9994	0.247	0.714	0.9905
	298	0.504±0.068	0.231	4.347	0.9992	0.085	0.654	0.9865
	318	2.406±0.022	1.532	0.650	0.9998	0.397	0.816	0.9911
MMIP	308	1.357±0.106	0.676	1.501	0.9990	0.240	0.705	0.9843
	298	0.351±0.005	0.175	6.048	0.9918	0.085	0.492	0.9489
	318	0.669±0.033	0.489	2.087	0.9920	0.171	0.618	0.9882
MNIP	308	0.351±0.006	0.276	3.759	0.9985	0.112	0.523	0.9904
	298	0.155±0.006	0.108	10.197	0.9896	0.058	0.389	0.9751

Table S5. Isolate factors β of HMMIP and MMIP

	benzoic acid	salicylic acid	2, 4-dihydroxybenzoic acid
HMMIP	3.684	3.593	5.230
MMIP	2.763	5.436	3.998
